# Sphingolipid Metabolism in Glioblastoma and Metastatic Brain Tumors: A Review of Sphingomyelinases and Sphingosine-1-Phosphate

**DOI:** 10.3390/biom10101357

**Published:** 2020-09-23

**Authors:** Cyntanna C. Hawkins, Tomader Ali, Sasanka Ramanadham, Anita B. Hjelmeland

**Affiliations:** 1 Department of Cell, Developmental, and Integrative Biology, University of Birmingham at Alabama, Birmingham, AL 35233, USA; cyntanna@uab.edu (C.C.H.); sramvem@uab.edu (S.R.); 2 Research Department, Imperial College London Diabetes Centre, Abu Dhabi P.O. Box 48338, UAE; tfali@icldc.ae; 3 Comprehensive Diabetes Center, University of Birmingham at Alabama, Birmingham, AL 35294, USA

**Keywords:** glioblastoma, sphingolipid, sphingosine-1-phosphate, sphingomyelinase, sphingomyelin, metastasis

## Abstract

Glioblastoma (GBM) is a primary malignant brain tumor with a dismal prognosis, partially due to our inability to completely remove and kill all GBM cells. Rapid tumor recurrence contributes to a median survival of only 15 months with the current standard of care which includes maximal surgical resection, radiation, and temozolomide (TMZ), a blood–brain barrier (BBB) penetrant chemotherapy. Radiation and TMZ cause sphingomyelinases (SMase) to hydrolyze sphingomyelins to generate ceramides, which induce apoptosis. However, cells can evade apoptosis by converting ceramides to sphingosine-1-phosphate (S1P). S1P has been implicated in a wide range of cancers including GBM. Upregulation of S1P has been linked to the proliferation and invasion of GBM and other cancers that display a propensity for brain metastasis. To mediate their biological effects, SMases and S1P modulate signaling via phospholipase C (PLC) and phospholipase D (PLD). In addition, both SMase and S1P may alter the integrity of the BBB leading to infiltration of tumor-promoting immune populations. SMase activity has been associated with tumor evasion of the immune system, while S1P creates a gradient for trafficking of innate and adaptive immune cells. This review will explore the role of sphingolipid metabolism and pharmacological interventions in GBM and metastatic brain tumors with a focus on SMase and S1P.

## 1. Introduction

**Figure 1:**
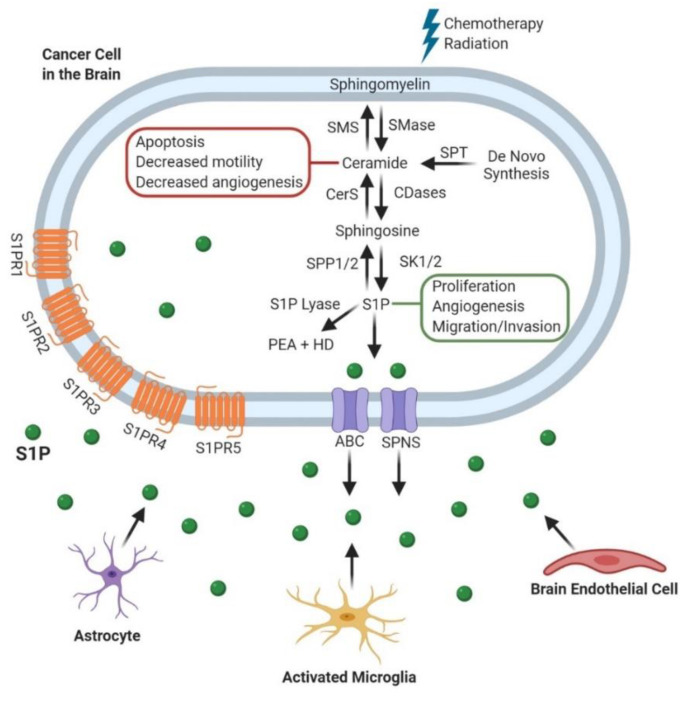
Sphingolipid Metabolism and its role in Cancer Progression. After chemotherapy and radiation, sphingomyelin is broken down into ceramide which has roles in blocking cancer progression. Cancer cells can convert ceramide to sphingosine-1-phosphate (S1P), which is transported out of the cell by either ATP-binding cassette (ABC) or spinster (SPNS) transporters [36,37]. S1P then exerts its pro-tumor effects through both intracellular and extracellular mechanisms. Alternatively, S1P can be degraded by S1P lyase to produce Phosphatidylethanolamine (PEA) and Hexadecenal (HD) [6,38]. These processes also occur in the other cell populations within the brain tumor microenvironment including astrocytes, microglia, and endothelial cells.Sphingomyelin Synthase (SMS); Ceramide synthase (CerS); Sphingosine phosphate phosphatase 1/2 (SPP1/2); Sphingomyelinase (SMase); Ceramidase (CDase); Sphingosine kinase 1/2 (SK1/2); Serine palmitoyltransferase (SPT); Sphingosine-1-phosphate (S1P); Phosphatidylethanolamine (PEA); Hexadecenal (HD); ATP-binding cassette (ABC); Spinster (SPNS).

In recent years, studies of the role of sphingolipid metabolism have become an integral part of cancer research. Sphingomyelins (SMs), predominant sphingophospholipids in the outer leaflet of cell membranes, and their hydrolysis by sphingomyelinases (SMase) are essential to the efficacy of chemo- and radiotherapy [1,2,3,4]. SMases are distinguished according to their subcellular location and optimal pH for activity: SMases are named based on the pH at which they are active, with acid SMase in the lysosome, neutral SMase at the plasma membrane, and alkaline SMase in the endoplasmic reticulum [5,6]. Activation of SMase results in the production of phosphorylcholine and a ceramide, the central lipid in sphingolipid metabolism [7]. Ceramide can also be produced by the salvage pathway (Figure 1). The salvage pathway and de novo synthesis involve ceramide synthases and serine palmitoyl transferase (SPT), respectively (See Gault et al. for a more detailed review of de novo synthesis) [8]. Ceramide has been linked to decreased cell motility and angiogenesis but is most well-characterized as a pro-apoptotic signal [9,10,11]. However, cells can escape apoptosis if ceramide is hydrolyzed by ceramidases (CDases) to sphingosine [7]. Like the SMases, the CDases are also distinguished by their subcellular location and optimum pH for activity: acid CDase, neutral CDase, and alkaline CDase [12,13,14]. The CDases catalyze cleavage of the fatty acid from ceramide to produce sphingosine, which can subsequently be phosphorylated by sphingosine kinases (SK1 and SK2) to generate sphingosine-1-phosphate (S1P) [8,15]. S1P is linked to increased cellular proliferation, angiogenesis, and motility [10,16,17,18]. The levels of ceramides and S1P can be modulated based on cellular stress through pathways described as a series of “drains” and “faucets” [19]. This has led to the concept of the sphingolipid rheostat, which illustrates the consequence of shifting the balance between ceramide (pro-apoptotic) and S1P (pro-proliferative) on cell survival [20,21].

In cancer, the sphingolipid rheostat tilts toward S1P, promoting cell signaling that increases survival, proliferation, and migration [20,22]. S1P signals through five G-protein coupled receptors designated S1P receptor 1-5 (S1PR1-5) by autocrine and paracrine mechanisms [23,24,25]. Initially referred to as endothelial differentiation genes (EDG), recognition of their ability to bind S1P prompted a name change to S1PRs (S1PR1/Edg-1, S1PR2/Edg-5, S1PR3/Edg-3, S1PR4/Edg-6, S1PR5/Edg-8) [26,27,28,29]. Each receptor can couple to different G-proteins based on their motifs with primary functions through G_i_, G_q_, and G_12_. Both G_i_ and G_12_ promote downstream effects through phospholipase C (PLC) and phospholipase D (PLD) [30,31,32]. PLC cleaves the proximal phosphodiester bond of glycerophospholipids to produce diacylglycerols and a phosphorylated headgroup, while PLD cleaves the distal phosphodiester bond to produce the headgroup and phosphatidic acid [33]. PLC can signal through protein kinase C to cause the intracellular release of Ca^2+^ and promotion of cell proliferation [34]. Additionally, phosphatidic acid produced by PLD can attract and bind SK1 at the plasma membrane where S1P is produced to fuel cell growth and survival [35].

The sphingolipid rheostat plays an important role in the progression of glioblastoma (GBM)—the most common primary malignant brain tumor. Patients with GBM have a median survival of only 15 months with standard of care, which includes maximum surgical resection, radiation, and chemotherapy with temozolomide (TMZ), and adjuvant TMZ [39,40]. Unfortunately, GBM recurs in almost all cases due to both the inability to remove tumor cells invading normal brain and development of therapeutic resistance. Contributing to the latter are a number of factors, including multiple facets of intra-tumoral heterogeneity, some of which involve a subset of cells called brain tumor-initiating cells (BTICs) [41,42]. The BTICs are a less-differentiated, neural stem cell-like population that preferentially survive chemo- and radiotherapy, propagate tumors in animal models, and are suggested to repopulate the tumor after therapeutic intervention [41,42,43,44]. Alterations in sphingolipid metabolism have recently been implicated in both differentiated GBM cells and the less differentiated BTICs [10,45]. Samples from GBM patients have shown an increase in S1P concurrent with a decrease in ceramides, as compared with normal brain, indicating tilting of the sphingolipid rheostat toward a pro-tumor phenotype [10]. Further, GBM cells grown in cell culture under conditions, which enrich for the BTIC population had even higher S1P compared to their differentiated GBM counterparts [45].

While brain tumors can arise from cells within the brain as in GBM, metastases of other cancers to the brain (including breast, colon, lung, and skin cancers) are more common causes of tumor development in the brain [46,47,48,49]. Altered sphingolipid metabolism is also evident in these cancers with a propensity for metastasis to the brain [50,51,52]. Metastasis to distant organs is characteristic of advanced stages of disease, with 10–30% of all cancer patients exhibiting brain metastasis [53]. Once brain metastasis is established, the disease becomes much more difficult to treat [51,54,55,56]. Although brain metastases vary in mutational load and immune system alterations from GBM, any advances that benefit GBM may also benefit metastatic cancers. This warrants further research of sphingolipid metabolism in the context of both GBM and metastatic brain tumors [57].

## 2. Sphingomyelinases (SMases)

Since sphingomyelinases are imperative to the efficacy of both radio- and chemotherapy, the study of their roles has provided novel information concerning treatment regiments and established new directions for research. Of particular interest is the modulation of cell death in patients resistant to radio- and chemotherapy which is common in both GBM and metastatic cancers, as discussed in earlier.

### 2.1. Glioblastoma (GBM)

Studies analyzing lung tissue from mice deficient for acid SMase show resistance to apoptosis following radiation and that the p53- and ceramide-induced apoptosis were distinct [58]. Additionally, others have shown that while p53 induction by irradiation can increase ceramides, induction of ceramide generation by other means does not always induce p53 expression in leukemia or fibroblast cell lines [59]. Initial studies showed that acid SMase overexpression sensitized glioma cells to chemotherapies, gemcitabine and doxorubicin [60]. In contrast, subsequent studies found that overexpression of acid SMase increased ceramide levels but failed to sensitize GBM cells to radiation or chemotherapy with TMZ, the current standard of care [61]. To control for differences in p53, GBM cell lines with mutant p53 expression or without p53 expression were assessed [62,63]. While acid SMase overexpression increased ceramide levels, the acid SMase overexpression may not have been high enough to sensitize cells to TMZ. The same study demonstrated that other methods of increasing ceramides, specifically a glucosylceramide synthase inhibitor or direct addition of C2- or C6-ceramide, decreased survival of TMZ-resistant glioma cell lines [61]. Acid SMase, but not neutral SMase, caused the hydrolysis of sphingomyelin to ceramides to induce apoptosis in p53-deficient GBM cells. The same study showed that wildtype p53 expression was capable of blocking the ceramide response by upregulating acid CDase to shift towards increased production of S1P from ceramides and allow the cells to evade apoptosis [64]. Conversely, the presence of p53 was able to generate ceramide through formation of reactive oxygen species and subsequent activation of neutral SMase [65]. Other studies suggest that p53 alters ceramide production by increasing alkaline CDase expression indicating a potential cell type-dependent mechanism [66]. The exact mechanism for the p53/ceramide interaction is not fully understood and continues to be an active area of investigation. Activation of neutral SMase in C6 glioma cells has been suggested to an increase in mitogen-activated protein kinase (MAPK) activation through upregulation of ceramides, leading to apoptosis [67]. Together, these data suggest that SMases can regulate ceramide levels and apoptosis in glioma cells, with differential responses, in part, due to p53.

### 2.2. Metastatic Cancers

Studies directly correlating SMases, expression or activity, to brain metastasis are limited. However, various studies suggest that SMase alterations can affect metastasis of cancers with higher propensities to metastasize to the brain. Human studies on non-small cell lung cancer demonstrated that these patients have increased acid SMase expression, which was suggested to be pro-tumorigenic via immunosuppression [68]. In a mouse model of melanoma, acid SMase deficiency showed prevention of lung metastasis by inhibition of secretory acid SMase in platelets. The metastatic phenotype was re-established when wildtype platelets were returned, again suggesting a pro-metastatic role for secreted acid SMase [69]. Conversely, high levels of sphingomyelins, which could suggest lower levels or activity of SMases, have been associated with a highly metastatic subset of prostate cancer cells [70]. Additional data in melanoma cells suggest that low acid SMase could be pro-tumorigenic via promotion of therapeutic resistance. Cells with low acid SMase expression display higher resistance to cisplatin, possibly due to the inability to produce ceramide [71]. Overexpression of acid SMase or addition of recombinant acid SMase to melanoma-bearing mice sensitized the tumors to irradiation. Additionally, in vitro culture of the B16 melanoma cells at a pH of 6.5 increased the activity of acid SMase at the cell membrane, suggesting that the pH of many solid tumors may increase the activity of acid SMase and increase radiosensitivity [72]. These studies suggest a role for acid SMase in metastasis and therapeutic resistance that may be cell- or level-dependent, making it an important enzyme to consider when looking at cancers that readily metastasize to the brain.

## 3. Sphingosine-1-Phosphate (S1P)

S1P has long been evaluated for its ability to promote tumor progression, but research in this context has been restrained by the availability of rigorous methods for quantification. However, as mass spectrometry approaches have improved, there has been an explosion of research into this ubiquitous sphingolipid species. This research advancement has created more questions than answers regarding its function, particularly concerning its signaling receptors. Both GBM and metastatic cancers have alterations in sphingolipid metabolism pushing them towards this pro-tumor species.

### 3.1. Glioblastoma (GBM)

S1P has been implicated in many of the aggressive phenotypes that arise in GBM. In patient samples of GBM, the sphingolipid rheostat is shifted toward S1P with a concurrent decrease in ceramides [10]. C18 ceramide showed the most dramatic decrease compared to C24:1 ceramide, C16 ceramide [10]. Abuhusain et al. showed that S1P concentration increased with tumor grade and led to an increase in angiogenesis with reported levels of 1 pmole/mg of tissue in GBM brain samples, compared to 0.2 pmole/mg of tissue in the normal brain samples. S1P has also been implicated in GBM invasion [73], with S1P upregulation of urokinase plasminogen activator as a possible mechanism [74]. The upregulation of S1P in GBM patients may be partially due to an upregulation of the enzymes that produce S1P in cell surviving radiotherapy [75,76]. Acid CDase and sphingosine kinase 1 (SK1), which shunt ceramide to S1P, were shown to be higher in GBM tissue compared to normal brain [10]. The acid CDase enzyme has also been associated with markers of the neural stem cell-like BTIC fraction [77]. Furthermore, extracellular rather than intracellular S1P, has been shown to promote survival of the BTIC population; with BTICs exporting more S1P than their differentiated GBM cell counterparts [78]. Marfia et al. subsequently reported that S1P could induce the proliferative effects of BTICs via autocrine signaling [45]. The extracellular S1P was also shown by Abdel Hadi et al. to be produced by brain endothelial cells in a co-culture model [79]. When considering the opposite side of the sphingolipid rheostat, the direct addition of C6-ceramide to GBM cells in culture induced apoptosis, further implicating the importance of ceramide and S1P ratio in cell fate decisions between apoptosis and survival [80].

Mechanistically, to initiate a cell signaling cascade, S1P binds to one of five different receptors (S1PR1-5). The S1PR1, S1PR2, and S1PR3 were shown to be elevated in patient brain tumor samples compared to normal brain, while S1PR4 was not expressed, and S1PR5 remained unchanged [76]. S1PR1-5 are critical for mediating different functions of S1P, but the direction in which they alter cellular phenotypes is not entirely clear (Table 1). For instance, inhibiting S1PR1 using siRNA had been reported to increase GBM proliferation, but conflicting studies suggested that signaling through S1PR1-3 all increase GBM proliferation with S1PR1 having the greatest effects [81,82]. For S1PR2, this receptor was reported to attenuate migration of GBM cells through the Rho kinase pathway, but was also involved in increasing invasion [83,84]. Studies from other patient tumor samples have implicated S1PR5 as an independent prognostic factor in GBM, which aligns with data that S1PR5 increased proliferation [82,85]. The discrepancies between Bien-Möller et al. and Quint et al. regarding S1PR5 expression is likely due to small samples sizes and the vast inter- and intratumoral heterogeneity exhibited in GBM [86]. Recent studies have investigated how S1P receptor levels and signaling may also be affected by the brain tumor microenvironment. Upon co-culture of GBM cells with brain endothelial cells, expression of S1PR1 and S1PR3 was elevated in GBM cells [79]. In the normal brain, S1P promoted the survival of mature oligodendrocytes through a protein kinase B (AKT)-dependent pathway, and S1PR5 was required for process retraction in immature oligodendrocytes, which is necessary during development [87]. Pharmacological alteration of S1PR expression by fingolimod, a sphingosine analog which leads to the internalization of S1PR1, also known as FTY720, decreased human astrocyte activation and altered C-X-C motif chemokine 5 (CXCL5) release from both astrocytes and microglia [88,89]. CXCL5 is known to increase proliferation and invasion in GBM cells, emphasizing the importance of this interaction with the tumor microenvironment [90]. These studies suggest the importance of S1PR signaling in brain tumor cells and the brain microenvironment, but additional understanding of biological consequences is needed to more fully predict the benefits and potential risks of S1PR modulation.

### 3.2. Metastatic Cancers

Metastatic cancers have been shown to produce and secrete more S1P, as compared to primary tumors [91]. Such secreted S1P has been reported to be capable of establishing pre-metastatic niches in distant organs, such as the brain, through mechanisms involving S1PR1 [91,92]. Inhibition of S1P signaling using fingolimod in multiple myeloma revealed that metastasis to the bone marrow was due to the C-X-C chemokine receptor 4 (CXCR4)/C-X-C motif chemokine 12 (CXCL12) pathway [92]. While metastasis specific to the brain has not been studied with respect to S1P, the expression of CXCL12 was positively correlated with brain metastasis in solid tumors [93]. This links CXCL12 and its downstream signaling pathways to brain metastasis.

For the remainder of this section, we will consider the broader role of S1P in metastasis with a focus on cancers with the highest propensity of metastasizing to the brain. However, we recognize that metastatic niches and molecular mediators of metastasis can vary by organ, and not all of the signals discussed here may be relevant for brain-specific metastasis. Of the breast cancer subtypes, triple-negative breast cancer (TNBC) has the highest and earliest likelihood of metastasizing to the brain [94]. Studies of TNBC report high expression of SK1 with a concurrent increase in S1P, which promotes growth through the S1P/S1PR3/Notch signaling pathway [95,96]. A study concerning obesity-related progression of breast cancer found S1P elevated in obese patients and mice [97]. S1P contributed to the establishment of a pre-metastatic niche, and targeting S1PR1 with fingolimod decreased metastasis to the lungs [97]. In non-small-cell lung carcinoma (NSCLC), elevated SK1 and SK2 mRNA expression was associated with a worse prognosis in patients, likely due to an increase in S1P produced by the cancer cells [98]. Zhao et al. further considered the role of S1P in NSCLC metastasis through S1PR3. When S1PR3 was either genetically or pharmacologically targeted, a decrease in metastasis was observed due to attenuation of the Transforming Growth Factor-β (TGF-β)/Mothers against decapentaplegic homolog 3 (SMAD3) signaling axis [99]. Studies in metastatic breast cancer showed a similar decrease in migration when inhibiting S1PR3, potentially through a S1P/S1PR3/Cyclooxygenase-2 (COX-2) pathway [100]. Paradoxically, chemotherapy itself may induce metastasis—one of the more serious complications of chemotherapy in solid tumors [101]. Inhibition of S1P signaling through S1PR1 was able to mitigate this serious side effect [102]. In contrast, JTE013, an antagonist of S1PR2, increased migration and invasion in melanoma, showing opposing signaling roles related to S1PR1 and S1PR3 [103]. Albeit less studied, S1PR4 was associated with a decrease in overall survival in estrogen receptor (ER)-negative breast cancer [104]. Beyond the S1PRs, overexpression of acid CDase in melanoma cells enhanced resistance to dacarbazine, the DNA-alkylating agent often used in patients [105]. Consistent with this finding for a pro-tumorigenic role of acid CDase, knockdown of acid CDase in melanoma cells decreased both growth and invasion [106]. Concerning the tumor microenvironment, S1P produced by higher SK1 expression was found to increase the differentiation of surrounding fibroblasts, further promoting metastasis of melanoma cells [107]. Lastly, a screen of over 800 mutant mice revealed that mice deficient in S1P transporter spinster homologue 2, the protein responsible for transporting S1P from the cell, had the least amount of pulmonary metastases [108]. Together, these data demonstrate critical roles for S1P/S1PR signaling in cancers that have a propensity to metastasize to the brain.

**Table 1:** Summary of the current research on S1PR effects. S1PRs have multiple effects on cancer cells, illustrating a lack of consensus on the predominant effects in all cancer types. Glioblastoma (GBM); triple-negative breast cancer (TNBC); non-small cell lung cancer (NSCLC)**.**

S1PR	Cancer Type	Alteration	Phenotype	Study
**S1PR1**	GBM	Absence	Increased proliferation	Yoshida et al., 2010 [81]
	GBM	Presence	Increased proliferation	Young et al., 2007 [82]
	Breast cancer	Decrease	Decreased metastasis	Nagahashi et al., 2018 [97]
	Solid tumors	Decrease	Decreased migration	Liu et al., 2015 [102]
**S1PR2**	GBM	Absence	Increased migration	Lepley et al., 2005 [83]; Malchinkkuu et al., 2008 [84]
	Melanoma	Decrease	Increased migration/invasion	Arikawa et al., 2003 [103]
**S1PR3**	GBM	Presence	Increased proliferation	Young et al., 2007 [82]
	TNBC	Presence	Increased metastasis	Wang et al., 2018 [96]
	Breast cancer	Decrease	Decreased migration	Filipenko et al., 2016 [100]
	NSCLC	Decrease	Decreased metastasis	Zhao et al., 2016 [99]
**S1PR4**	Breast cancer	Presence	Decreased survival	Ohotski et al., 2012 [104]
**S1PR5**	GBM	Presence	Increased proliferation	Young et al., 2007 [82]

## 4. Phospholipase-Mediated Signaling

One of the predominant ways that S1P can signal within the cell is through phospholipase-mediated pathways. Phospholipases C (PLC) and D (PLD) have emerged as major contributors to the aggressive phenotypes seen in GBM including invasion and chemoresistance, as well as promotion of metastasis in other cancer types [109,110,111,112]. The intersection between sphingolipid metabolism and phospholipase signaling provides a greater understanding of how these pathways synergize to promote cancer progression in both GBM and metastatic cancers.

### 4.1. Glioblastoma (GBM)

**Figure 2:**
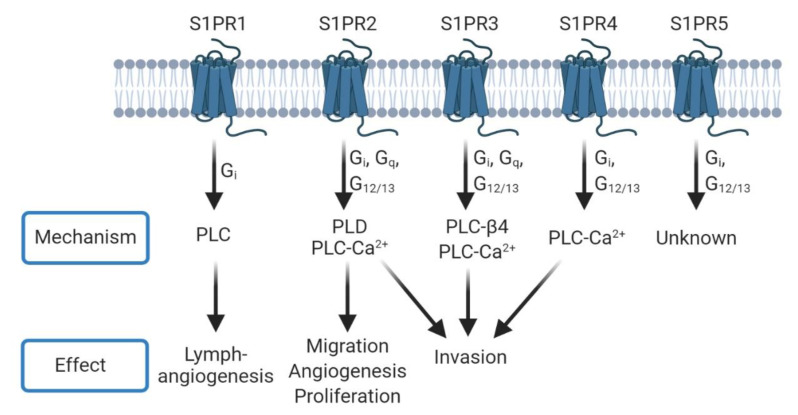
S1PRs can signal through phospholipase mechanisms. Each S1PR can couple to one or more G Protein-Coupled Receptors (GPCRs) to signal through different phospholipases and induce phenotypes such as angiogenesis, proliferation, and invasion. Many of these mechanisms overlap between receptors. Conversely, PLD signaling can lead to further production of S1P through the interaction of phosphatidic acid with sphingosine kinase 1 (SK1) [35].

Signaling through the S1P receptors can alter phospholipase signaling, particularly that of PLC (Figure 2). Addition of S1P induced activation of matrix metalloproteinase-9 (MMP-9), which is known to increase invasion in breast cancer cells. S1P exerted its effects through Gαq and S1PR3, and this further induced the expression of PLC-β_4_ (PLC-β_4_) via Rac1 [113]. In other studies, MMP-9 expression was reported to be increased at an extracellular acidic pH (5.4–6.5), which is common in the GBM tumor microenvironment [114]. Later studies from the same group reported induction of SMase by extracellular acidic pH mediated MMP-9 activity, through nuclear factor kappa-light-chain-enhancer of activated B cells (NF-κB) activation [115]. In C6 rat glioma cells, activation of S1PR2 led to downstream signaling via the PLC-Ca^2+^ system, as well as PLD. This further activated extracellular-signal-regulated kinase (ERK), which can stimulate proliferation, migration, and angiogenesis [116]. Notably, only S1PR1 and S1PR2 were expressed on C6 glioma cells, which is not consistent with the reported expression in glioma patient samples [76]. Overall, these findings suggest the importance of understanding S1P-mediated phospholipase signaling in order to elucidate the mechanisms behind GBM progression.

### 4.2. Metastatic Cancers

The interaction between sphingolipid metabolism and phospholipase signaling in metastatic cancers extends our mechanistic insight into GBM pathology. Recent investigations show S1P can trigger the release of Ca^2+^ to increase phosphatidic acid through activation of PLD [117]. Phenotypically, S1P can promote the formation of lymphatic vessels [118]. This process, called lymphangiogenesis, is thought to be similar to angiogenesis, occurs in cancer, and is associated with lymph node metastasis [119,120]. Using human lymphatic endothelial cells (HLECs) and in vivo models, Yoon et al. found that S1P promoted lymphangiogenesis through S1PR1 and phospholipase C [118]. In lung adenocarcinoma cells, S1P added in vitro increased the activity of phospholipase D, leading to a dramatic increase in RhoA [121]. The induction of RhoA as part of signaling through PLCε and G Protein-Coupled Receptors (GPCRs) with S1P treatment has also been shown in astrocytes as part of a neuroinflammation model [122]. To date, there is very limited knowledge on the exact convergence of sphingolipid metabolism and phospholipase signaling in metastatic cancers, particularly to the brain, but investigations in other cancers and of the pathways independently suggest that this will be a very active area of exploration in upcoming years.

## 5. Blood–Brain Barrier Integrity

**Figure 3:**
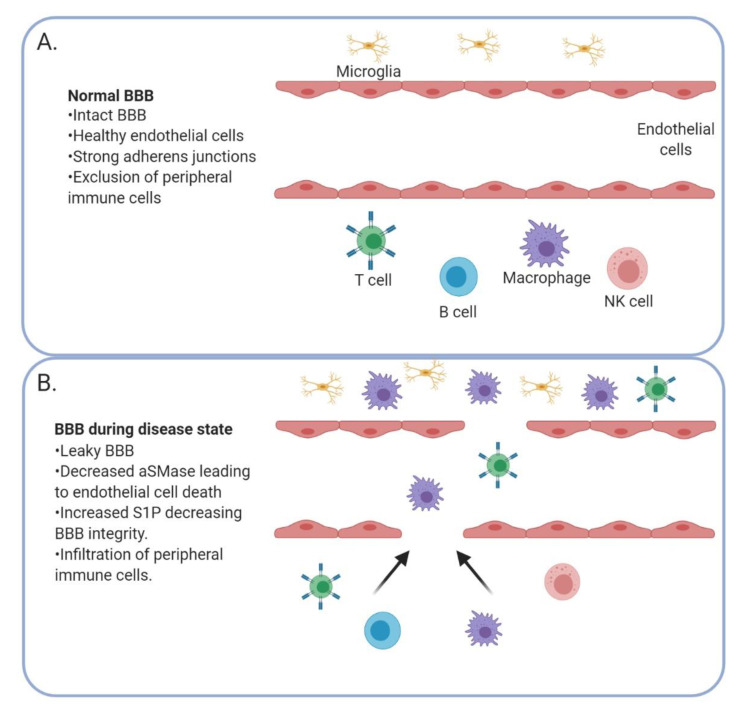
The blood–brain barrier (BBB) is altered in the context of a brain tumor. A healthy BBB (**A**) with strong adherens junctions prevents peripheral immune cells from entering the brain. When the BBB is compromised (**B**) by a decrease in acid SMase and increase in S1P, peripheral immune cells can enter the brain.

The blood–brain barrier (BBB) consists of endothelial cells connected by tight junctions and protects the brain by preventing invasion of deleterious molecules present in circulation [123]. The BBB also prevents the passage of many cancer therapeutics, precluding a large number of drugs from effectively targeting GBM or metastatic brain tumors. While there are leaky areas of the BBB in a tumor-setting, they are not evenly dispersed throughout, so not all portions of the brain tumor will receive the drug uniformly (Figure 3). As GBM has been suggested to be a “whole brain disease,” failure to target even a small section of the tumor or the invading tumor cells will lead to recurrence of the disease [124]. During aging, the BBB begins to deteriorate and correlates with elevated acid SMase levels in the plasma of humans. Park et al. recapitulated this finding in an aged mouse model and determined that increases in acid SMase led to endothelial cell death and disruptions in the BBB via altering caveolae internalization [125]. Apoptosis of endothelial cells post-irradiation in a rat model also caused breakdown of the BBB through an acid SMase-dependent mechanism [126].

Furthermore, the role of S1P signaling in BBB integrity has become an interesting avenue of research [127]. A study of ischemia-reperfusion injury demonstrated that S1P increased STAT3 activation, leading to BBB dysfunction [128]; these data suggested that inhibition of S1P could be a strategy to prevent BBB breakdown. However, knockdown of S1P lyase (an enzyme that breaks down S1P) in endothelial cells of the BBB increased expression of adherens junction molecules, leading to increased BBB integrity in vitro [129]. Thus, inhibition of S1P lyase may prevent the breakdown of the BBB caused by inflammatory factors [129]. One explanation of the discrepancies in S1P effects on the BBB was provided by Li et al. using human umbilical vein endothelial cells (HUVECs) in culture: they showed that physiologic concentrations of S1P promoted assembly of tight junctions via S1PR1 and Rac1 activation, but higher concentrations actually led to the disassembly of tight junctions via S1PR2 and the RhoA/ROCK pathway [130]. Additional work by van Doorn et al. found that expression of S1PR5 on brain endothelial cells was crucial for maintaining BBB integrity [131]. Additionally, a study utilizing co-cultured endothelial cells and astrocytes to mimic the BBB has observed that fingolimod increased endothelial cell survival when exposed to inflammatory cytokines by inducing the release of Granulocyte-macrophage Colony-Stimulating Factor (GM-CSF) from astrocytes [132].

Emerging therapies for GBM are exploring ways to open the BBB in order to allow anti-cancer therapies to cross the BBB. For instance, a clinical trial (NCT03712293) where patients received standard of care and magnetic resonance-guided focused ultrasound to disrupt the BBB proved to be safe and accurate and such trials are continuing [133]. Other in vivo studies demonstrated that pharmacologic inhibition of S1PR1 allowed for transient opening of the BBB by altering tight junction protein localization [134]. These reports open the door for many new treatments in GBM and metastatic brain tumors, but the risks of disrupting the BBB must be considered, particularly if it cannot be reliably restored post-treatment.

## 6. Immune Trafficking

In recent years, research into cancer immunology has focused on activating the patients’ immune system for tumor elimination, including checkpoint inhibitors to alter the adaptive immune response. While checkpoint inhibitors have largely failed in GBM, they have shown promising results in cancers with higher mutational loads such as melanoma [135]. Much of the research in GBM immunotherapy has focused on altering the innate immune system [136]. Sphingolipid metabolism plays a role in the trafficking of both adaptive and innate immune cells.

### 6.1. Adaptive Immunity

Multiple components of sphingolipid metabolism have been shown to regulate adaptive immunity, which is characterized by T and B cell responses days after infection [137]. Under normal conditions, there are very few, if any, T-cells that cross the BBB. During neurological disease, the breakdown of the BBB allows T-cells, among others, to enter the brain [123,138]. Compared to GBM, metastatic brain tumors have a substantially higher number of infiltrating lymphocytes [139]. While this process could lead to the elimination of a tumor, it often allows tumor-promoting populations to enter the brain such as regulatory T-cells (Tregs). which are CD4^+^CD25^+^FoxP3^+^ [140]. Tregs serve to protect the host from autoimmune disorder, but in cancer, they can promote tumor growth through their immunosuppressive functions [141]. Interestingly, acid SMase-mediated activation has been linked to CD4^+^ T-cells proliferation [142]. Further, mice deficient in acid SMase demonstrate an increase in Tregs globally, indicating a shift toward the immunosuppressive phenotype [143]. However, an increase in activated CD4^+^ and CD8^+^ T-cells with no change in Tregs was seen when NSCLC cells were injected into acid SMase deficient mice [68]. These discrepancies highlight the importance of the tumor microenvironment and the differential effects of acid SMase expression by the tumor cells, as compared to the immune cells.

S1P has long been appreciated for its ability to create a gradient for T-cell trafficking. Recent studies demonstrated that the presence of a brain tumor in mice, whether primary or metastatic, causes downregulation of S1PR1 on the surface of T cells, and leads to the homing and sequestration of T-cells in bone marrow [144]. This downregulation of S1PR1 has been exploited for the treatment of multiple sclerosis using fingolimod. Fingolimod is a sphingosine analog; it acts as a functional antagonist of S1PR1 by the phosphorylation of FTY720 to FTY720-Pi, alleviating the symptoms of autoimmunity [145]. This double-edged sword of S1P makes targeting the pro-proliferative and migratory lipid challenging. In the context of multiple sclerosis, blocking immune trafficking leads to a decrease in disease severity [145,146]. However, the same effect is not beneficial to brain tumor patients who already exhibit lymphopenia [144]. Conversely, van der Weyden et al. showed that mice deficient in the S1P transporter spinster homologue 2, the protein responsible for transporting S1P from the cell, had an increase in T cells and natural killer cells in the lung preventing metastasis [108]. This research suggests site-specific roles of S1P and the regulation of immune trafficking, revealing an exciting new area of investigation in GBM and metastatic cancers.

### 6.2. Innate Immunity

Sphingolipid metabolism contributes to the innate immune system, which provides rapid defense against foreign bodies and involves multiple cell types, including macrophages, dendritic cells, mast cells, and granulocytes. In GBM, macrophages constitute up to 50% of the bulk tumor, making them the primary innate immune cell in the tumor microenvironment [147,148]. In simplest terms, macrophages can be described as classically (M1) or alternatively activated (M2) with M1 being proinflammatory and M2 being the immunosuppressive and pro-tumorigenic tumor-associated macrophages (TAMs) [149]. Immunosuppressive TAMs express inducible nitric oxide synthase (iNOS), which can produce nitric oxide (NO) and lead to resistance of cancer cells to cisplatin—a commonly used chemotherapeutic agent. The NO induction also decreased acid SMase in glioma cells, allowing them to escape apoptosis [150]. Studies using melanoma cell lines demonstrated that low aSMase expression contributes to a pro-tumor immune response by allowing myeloid-derived suppressor cell accumulation [151].

**Figure 4:**
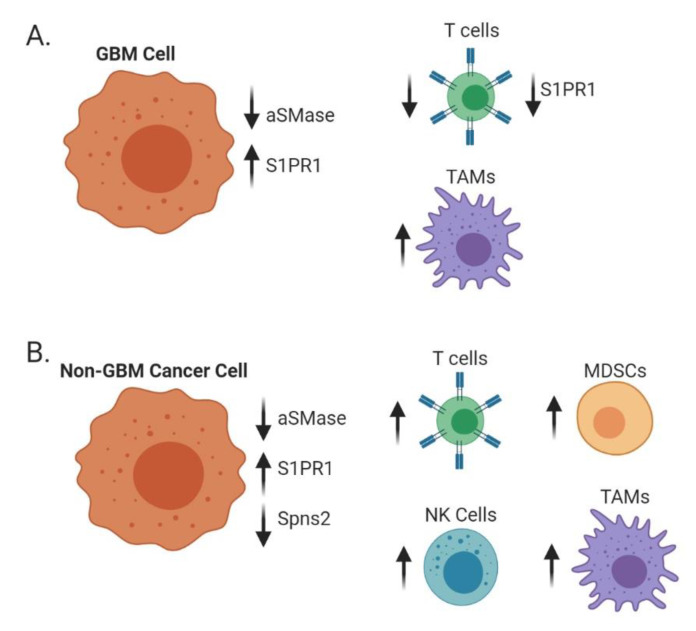
Alterations of sphingolipid metabolism affect surrounding immune populations. In GBM cells (**A**), low acid SMase and high S1PR1 on GBM cells but low S1PR1 on T cells decrease T cell recruitment while increasing macrophage recruitment and polarization towards an immunosuppressive (M2) phenotype. In metastatic brain cancers (**B**), such as melanoma, breast, and lung, decreases in acid SMase and Spns2 with increases in S1PR1 can increase recruitment and activation of both adaptive and innate immune cells.

As a result of apoptosis, expression of SK1 leading to S1P production has been shown to be a chemoattractant for macrophages [152]. In physiologically normal cells, this process is beneficial as migrating macrophages arrive to clear the waste of dying cells. Unfortunately, when SK1 is increased as a response to chemo- or radiotherapy, macrophages that are recruited to the tumor microenvironment can shift towards immunosuppressive phenotypes and promote tumor growth [10,153]. To this point, melanomas that express high levels of SK1 have a greater infiltration of macrophages and polarization to the immunosuppressive phenotype [154]. In fact, the expression of S1PRs on the surface of macrophages can be altered under different polarizing conditions in vitro. For example, M1 polarized macrophages had decreased expression of S1PR4 compared to unpolarized bone marrow-derived macrophages. In contrast, S1P1R was decreased in both M1 and M2 polarized macrophages in comparison to unpolarized bone marrow-derived macrophages. Differential downregulation of S1PRs with polarization could suggest that S1P activity alters macrophage biology. While S1P did not alter the phagocytic activity [155], deletion of S1PR1 on macrophages prevented pulmonary metastasis and lymphangiogenesis. This effect was mediated through attenuation of Nucleotide-Binding Oligomerization Domain, Leucine-Rich Repeat and Pyrin Domain Containing 3 (NLRP3) expression and interleukin 1-β (IL-1β) production in a breast cancer mouse model [156]. Interleukin 22 Receptor 1 (IL-22R1) signaling through S1PR1 also leads to the recruitment of macrophages to the tumor microenvironment in breast cancer [157]. As discussed above, fingolimod can also have an effect on the myeloid-derived suppressor cells (MDSCs). The MDSCs are a pro-tumorigenic immune population that accumulate in tissues when mice were treated with fingolimod. In long-term usage, this was shown to increase the risk of cancer development [158]. Subsequent investigations revealed that fingolimod decreased recruitment of macrophages to the brain tumor microenvironment, as well as pushed them toward a proinflammatory (M1) phenotype via C-X-C Motif Chemokine Receptor 4 (CXCR4) internalization [159]. Outside of the tumor setting, mice without S1P lyase expression had greater microglial activation in the brain. The accumulation of S1P signaled through S1PR2 to mediate this inflammation [160]. Figure 4 shows an overview of the alterations in both the adaptive and innate immune cells in GBM and metastatic cancers.

## 7. Altering Sphingolipid Metabolism for Therapeutic Intervention

As research continues into the role of sphingolipid metabolism, many new and repurposed therapeutics have emerged to modulate this pathway and improve the survival of patients with GBM as well as metastatic brain cancers [161]. Many of these emerging therapies are already in clinical trials, but there are still many unanswered questions regarding the efficacy of these treatments.

### 7.1. Glioblastoma

While the BBB serves as a barrier to many GBM therapies, other research efforts have focused on restoring the BBB to prevent the recruitment of immune suppressive cell populations. Drugs that target acid SMase, such as amitriptyline hydrochloride, have the potential to block the progression of both GBM and metastatic cancers by restoring the BBB [133]. However, that same inhibition of acid SMase can potentially decrease the efficacy of chemo- and radiotherapy. Thus, when considering combinatorial therapies, timing can prove to be very important. The small molecule inhibitor ARC39 has shown specificity of acid SMase in vitro, as well [162]. However, this would likely blunt the effects of chemo- and radiotherapy as they partially rely on the breakdown of sphingomyelin by SMases to induce apoptosis. The majority of therapeutic focus in this area has been on enzymes involved in S1P production, although some have suggested that vitamin D metabolites could activate the sphingomyelin pathway [163]. Inhibitors of SK1 have shown mixed results in GBM with one study showing that selective SK1 inhibitors, SKI-1a and SKI-1b, did not affect cell death but instead blocked angiogenesis, while others have shown that SK1 inhibition using SKI-II could be more effective than TMZ due to the induction of reactive oxygen species [10,164]. This difference in findings could be due to the selectivity of the inhibitors as SKI-II targets both SK1 and SK2 making SKI-II more efficacious at decreasing S1P production [165]. Additionally, the study that showed no difference in cell death used concentrations up to 1 µM, while the study that showed cell death used up to 20 µM, a 20-fold difference in concentrations. Others have shown that inhibitors of SK1 have efficacy in GBM cells which are TMZ-resistant [166]. Within the conflicting reports, some have suggested SK inhibitors be added as a maintenance therapy to prevent S1P formation and sustain ceramide induction [167]. In GBM, epidermal growth factor receptor (EGFR) is often mutated or constitutively active. EGFR inhibitors have also been shown to decrease activation of SK1, consequentially leading to a decrease in S1P and invasion [168]. Growth of GBM cells in vitro can be inhibited by acid CDase inhibitors such as carmofur [77,169]. Additionally, tamoxifen, a treatment for ER-positive breast cancer, has been shown to inhibit acid CDase and readily crosses the BBB [170]. As an already approved therapy, this provides potential application for the treatment of GBM. Other inhibitors of acid CDase such as B13 and the LCL series of compounds have yet to be tested in clinic despite showing promise in mouse models of prostate cancer [171,172]. One reason they have not been investigated further in GBM is that their structures make them unlikely to cross the BBB. Lastly, fenretinide (4-HPR), a synthetic retinoid with an early FDA approval for T-cell lymphoma, has been investigated for its ability to increase ceramide production by p53-independent mechanisms in cancer cells [173,174,175,176]. While 4-HPR has shown promise in vitro, its poor solubility and bioavailability have limited its use clinically, with both of the glioma clinical trials showing no improvement in progression-free survival at the doses administered (Table 2) [175,177].

Intriguingly, there have been reports of multiple sclerosis patients treated with fingolimod that later developed high grade gliomas [178]. The mechanism behind this etiology is unclear, but possibly linked to the immunosuppression or activation of additional S1P receptors. These alternative effects have generated interest in developing drugs that target specific S1P receptors in order to mitigate the effect on T-cell trafficking or tumor cell migration. Studies in multiple sclerosis using fingolimod as a functional antagonist of S1PR1 have indicated that it does not alter BBB integrity nor change MMP-9 expression [179]. These data indicate that fingolimod may not function through the same mechanisms as endogenous S1P [179]. Additionally, phosphorylated fingolimod was reported to bind to S1PR3, S1PR4, and S1PR5 as well as S1PR1, the receptor primarily responsible for immune trafficking [180]. Since the drug is phosphorylated by SK2 only, it can act as a SK1 inhibitor as demonstrated in prostate cancer [181,182]. Nonetheless, due to favorable toxicity profile of fingolimod, it has been clinically evaluated for safety in combination with radiation and TMZ in glioma patients (NCT02490930). Interestingly, fingolimod was given one week prior to radio- and chemotherapy with the hope of sequestering lymphocytes away from systemic circulation, thus protecting them from the immunosuppressive effects of radiation. The final results of that study are not yet available. As a whole, there are many promising avenues for new therapeutics in GBM patients, particularly as more specific inhibitors to enzymes in this pathway are discovered. Subsequently, a more thorough understanding of sphingolipid metabolism will provide more information for therapeutic design.

### 7.2. Metastatic Cancers

Developments in GBM treatments may translate to metastatic brain cancers and, thereby, open the door to more effective treatment in advanced stages of other cancers. A study in TNBC found that a nanoparticle containing docetaxel and fingolimod could abrogate lymphopenia while still blocking progression [183]. Carmofur, a derivative of 5′ fluorouracil and an inhibitor of acid CDase, has been used as adjuvant therapy in colorectal cancer patients to prevent metastasis [184]. The drug, which is known to cross the BBB, has been used for years in Japan as maintenance therapy following standard of care but has not been subjected to FDA approval in the United States [185]. While relatively rare, carmofur has been linked to cases of leukoencephalopathy in patients [186]. This side effect is likely one of the reasons it has not been approved for use in the United States. It is unclear whether this is a side effect of the sphingolipid metabolism alterations or an off-target effect of carmofur. A study of hispidulin, a polyphenolic flavonoid, found that the treatment shifted the sphingolipid rheostat to ceramide by inhibiting SK1 [187]. ABC294640 is the first-in-class inhibitor of SK2 and has been clinically tested in a variety of cancers (Table 2) [188]. ABC294640 has proven effective in both in vitro and in vivo models of TNBC [189]. While efficacy has yet to be determined in clinic, the treatment had minimal side effects in a phase I clinical trial (NCT01488513). The ability to repurpose already known modulators of sphingolipid metabolism may serve as exciting new treatments in metastatic cancers. Additionally, advancements in GBM therapeutics are likely to translate to metastatic brain cancers. Unfortunately, it is still unknown if many of these novel modulators of sphingolipid metabolism are able to cross the BBB, reinforcing the need for further investigation of these treatments for GBM and metastatic brain cancers.

## 8. Conclusions

Research in the field of sphingolipid metabolism has grown exponentially recently. This is particularly so with regards to the most aggressive cancers including GBM and cancers that have metastasized to the brain such as breast and lung cancers. As experimental methodologies and sphingolipid analysis techniques continue to advance, more therapeutically targeted approaches with more specific and efficacious treatments become feasible. Continuing to elucidate the evidently complex mechanistic interplay of the various molecules involved, and the resultant physiological implications, is imperative for the enhancement of cancer research and for working towards overall positive clinical outcomes.

## Figures and Tables

**Table 2:** Current and previous clinical trials targeting sphingolipid metabolism. Multiple clinical trials have attempted to target sphingolipid metabolism by altering enzymes, receptor expression, and ceramide accumulation in the sphingolipid pathway.

Cancer Type	NCT Number	Dates	Drug Name	Primary Target	Phase	Results	FDA Approval?
**GBM**	NCT02490930	July 2015–September 2017	Fingolimod	S1PR1 antagonist	Early Phase 1	Not Published	Yes
**Recurrent Glioma**	NCT00006080	September 2000–September 2004	Fenretinide (Single Agent)	Ceramide	Phase 2	Ineffective at the doses given	Yes
**Recurrent GBM**	NCT00075491	December 2003–March 2005	Fenretinide (Combination treatment)	Ceramide	Phase 2	Not Published due to termination	Yes
**Advanced solid tumors**	NCT01488513	August 2014–August 2015	ABC294640	SK inhibitor	Phase 1	Tolerated up to 500mg bid	No
**Advanced solid tumors**	NCT02834611	March 2017–August 2019	Ceramide NanoLiposome	Ceramide	Phase 1	Not Published	No
